# Topology Determines DNA Origami Diffusion in Intestinal Mucus

**DOI:** 10.1021/acs.nanolett.5c02352

**Published:** 2025-08-28

**Authors:** Matteo Tollemeto, Lars J. M. M. Paffen, Lasse H. E. Thamdrup, Anja Boisen, Jan van Hest, Tania Patiño Padial

**Affiliations:** † Department of Biomedical Engineering, Institute for Complex Molecular Systems, 3169 Eindhoven University of Technology, Het Kranenveld 14, 5600MB Eindhoven, The Netherlands; ‡ The Danish National Research Foundation and Villum Foundation’s Center IDUN, Department of Health Technology, 5205 Technical University of Denmark, DK-2800 Kgs. Lyngby, Denmark

**Keywords:** DNA origami, mucosal barriers, nanomedicine, single-particle tracking

## Abstract

Efficient nanomedicine delivery across mucosal barriers remains a challenge due to the complex and poorly understood relationship between nanoparticle design and mucus transport. Here, we present DNA origami as a platform to investigate how the nanoparticle shape and ligand patterning influence diffusivity in mucus. By decoupling these parameters while maintaining identical material composition, we systematically evaluated the diffusion of rod, icosahedral, and rectangular nanostructures by using high-resolution single-particle tracking. Our results reveal that diffusivity in mucus is not solely determined by shape or functionalization alone but by their interplay: while unmodified rods diffused poorly, their mobility increased significantly upon antibody functionalization, reaching a maximum at an intermediate ligand density. In contrast, rods and icosahedra exhibited less pronounced and nonoptimal responses to surface modification. These findings highlight the importance of topology-specific optimization in nanoparticle design and demonstrate the utility of DNA nanotechnology to uncover design rules for next-generation mucus-penetrating drug delivery systems.

## Graphical abstract

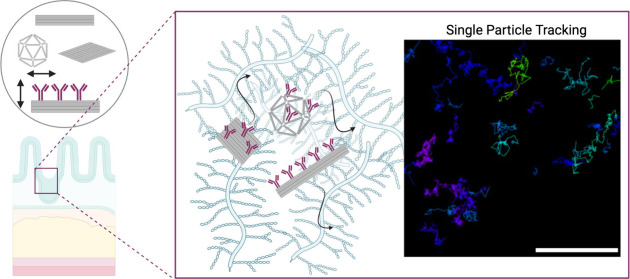

Nanomedicines have emerged as a promising strategy for enhancing the bioavailability of drugs by improving their stability, solubility, and accumulation at specific target locations. However, biological barriers continue to present major challenges for the efficient and targeted delivery of nanoparticles.^1–3^ One important barrier is the mucus layer that lines various epithelial surfaces, including those in the respiratory and gastrointestinal tracts.^4^ Mucus is a complex biological medium composed of water, mucins (glycoproteins), DNA, proteins, lipids, and cell debris, and its primary function is to lubricate and serve as a protective shield to exposed epithelia. The natural protective role of mucus presents a significant obstacle to nanomedicine transport due to its complex structure and high viscosity. In this regard, not only does the mucus barrier act as a physical impediment but also it influences the diffusion and absorption of nanoparticles, ultimately affecting therapeutic outcomes.^5,6^ While there are numerous examples of mucus-penetrating nanoparticles for drug delivery,^7,8^ the mechanisms governing nanoparticle behavior within mucus under varying physiological and pathophysiological conditions remain insufficiently understood. Several studies have shown that factors such as size,^9^ shape,^10^ surface properties,^11,12^ and rigidity^13^ significantly affect nanoparticle diffusion through intestinal mucus. Despite significant advances in mucosal drug delivery, understanding how nanoparticles such as size, shape, and ligand presentation influence diffusion through mucus remains a major challenge. Existing nanoparticle systems often lack the modularity and precision needed to independently vary these parameters without altering other critical properties, such as surface chemistry or material composition.

In this context, DNA origami offers a uniquely programmable nanofabrication strategy that enables the construction of 1D, 2D,^14^ and 3D^15–17^ nanostructures with precise control over geometry and uniform material composition. This can be achieved by the folding of an ssDNA scaffold using shorter compatible DNA strands (staples). Due to its unique programmability and design tunability while keeping the same material properties, DNA origami is emerging as a valuable platform for studying and optimizing nanoparticle interactions with biological systems.^18–27^ While DNA origami has been extensively used in other areas of nanomedicine, its application to systematically investigate the role of nanoparticle topology in mucus diffusion remains largely unexplored. In this work, we leverage DNA origami’s structural versatility to design a panel of topologically distinct nanoparticles and assess their transport behavior in mucus, enabling mechanistic insights that are difficult to obtain with conventional nanoplatforms. For this, we employed three distinct DNA-origami shapes, i.e., icosahedron, rectangle, and rod, and we functionalized them with the therapeutic anti-EGFR (Epidermal Growth Factor Receptor) antibody, given its potential for colorectal cancer applications.^28–30^ To investigate the diffusion dynamics of DNA origami within mucus in space and time, we employed single-particle tracking (SPT), a high-resolution optical microscopy technique that allows for the tracking of individual nanostructures, including DNA origami^31,32^ with unique spatiotemporal resolution.

By harnessing the precise and programmable design features of DNA origami, we provide a systematic approach to understanding how nanostructure design impacts mucus penetration. This study offers insights that could aid in the rational design of more effective drug delivery systems, contributing to improved bioavailability and therapeutic efficacy.

## DNA Origami Design, Assembly, and Characterization

One commonly recognized criterion for achieving optimal diffusion across mucus layers is the size of the nanoparticles, with those below 200 nm generally considered most effective.^33,34^ This size range allows nanoparticles to navigate through the dense meshwork of mucin present in mucus, enhancing their ability to penetrate mucosal barriers. Therefore, when designing the shapes and particles for our study, we specifically selected DNA origami structures that align with this criterion, where all DNA origami structures (i.e., rods, rectangles, and icosahedrons) were below the 200 nm threshold. The rod, rectangle, and icosahedron geometries were intentionally chosen to represent three distinct topological classes with increasing degrees of symmetry and dimensional complexity: (i) a high-aspect-ratio 1D structure (rod), (ii) a flat 2D geometry (rectangle), and (iii) a compact 3D polyhedron (icosahedron).^35,36^ This range allowed us to systematically investigate how particle dimensionality and surface curvature influence diffusion through mucus while maintaining comparable overall dimensions. These nanostructures were constructed through bottom-up self-assembly involving the folding of a long single-stranded DNA (ssDNA) scaffold. For the rods, the scaffold utilized was p7560, while for rectangles and icosahedra, the M13mp18 scaffold was employed. The assembly process was achieved by employing multiple ssDNA strands complementary to specific regions of the scaffold (i.e., ssDNA staple strands). Both the ssDNA scaffold and strands were mixed and subjected to thermal annealing, allowing the nanostructures to self-assemble through complementary base pairing interactions (Figure 1A). Gel electrophoresis was utilized to confirm the self-assembly of the scaffold into the three different shapes, where it was observed how the folded structures migrate differently compared with the scaffold. For electrophoretic analysis, unfolded scaffold strands (M13mp18, 7249 nt; P7560, 7560 nt) were used as internal size references in place of standard DNA ladders. The electrophoretic mobility of each structure was consistent with successful folding, as evidenced by distinct retarded bands relative to the scaffold. These shifts reflect the increase in the size and double-stranded content of the assembled nanostructures. Additionally, the lack of lower bands associated with excess staples confirms the effective purification via PEG precipitation. No significant residual oligonucleotide content was observed, supporting that the folded structures were obtained with high purity (Figure 1B). Next, we characterized the hydrodynamic size of rod, rectangle, and icosahedron designs by DLS, which displayed hydrodynamic diameters of 161.8 ± 6.1, 92.7 ± 5.6, and 95.1 ± 4.8 nm, respectively, with a PDI of 0.3 for the rods, 0.19 for the rectangles, and 0.18 for the icosahedra (Figure SI 1). To further confirm the size and shape of the folded DNA origami nanostructures, we performed AFM imaging. The average size was calculated by measuring 50 DNA origami for each particle shape, where a size of 142.6 ± 2.7 and 23.5 ± 2.4 (height × diameter) for the rods, 94.2 ± 11.7 and 66.8 ± 4.0 (height × length) for the rectangles, and 72.7 ± 7.2 nm in diameter for the icosahedra was observed (Figure 1C). These measurements are consistent with literature values, indicating dimensions of approximately 140–150 nm × 20–30 nm for the rods, 90 nm × 70 nm × 2 nm for the rectangles, and 50 nm diameter for the icosahedrons.^37–40^

**Figure 1:**
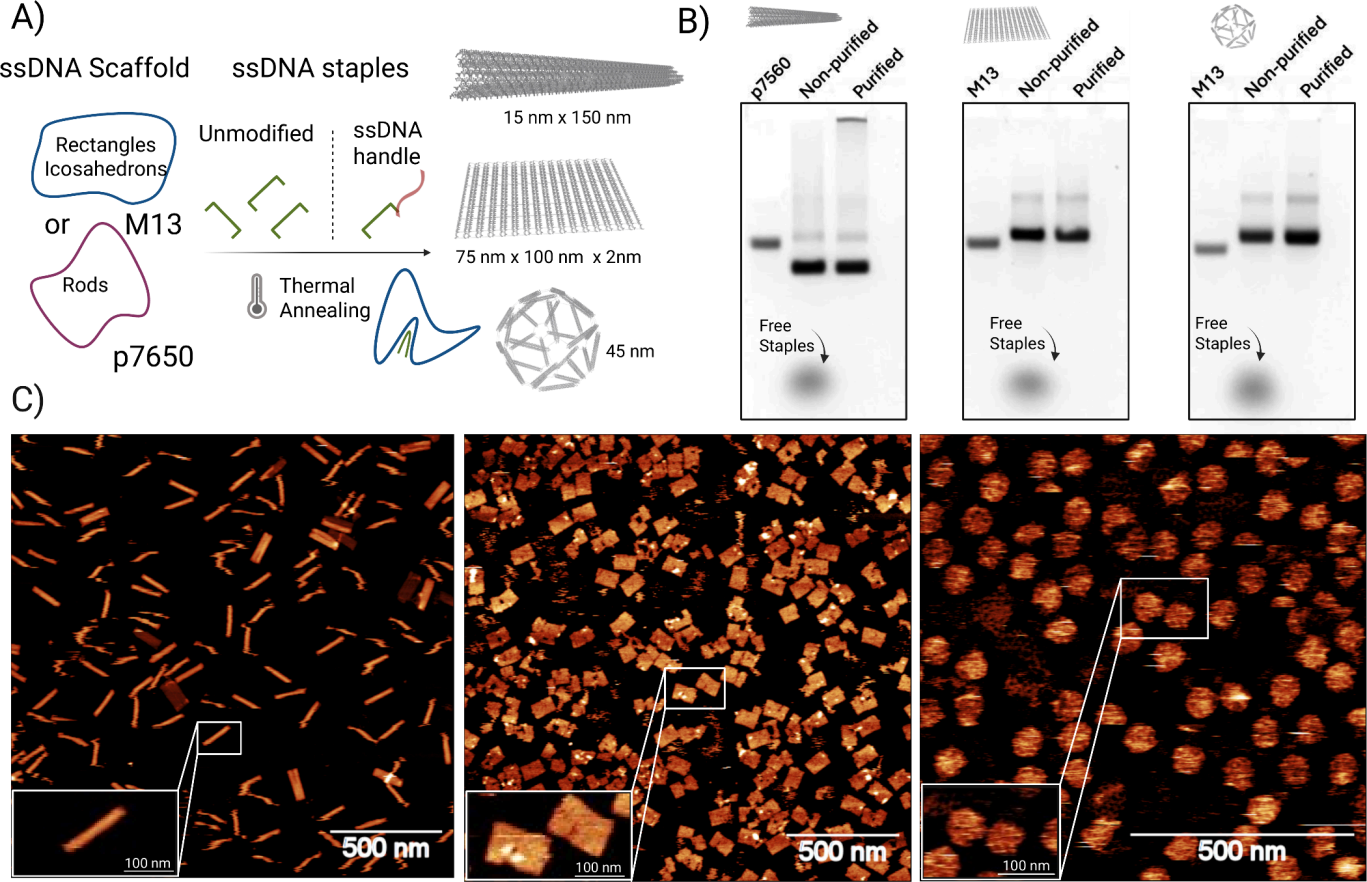
DNA origami assembly. A) Illustrative diagrams depicting the assembly of DNA origami into various shapes. B) Agarose gel electrophoresis of DNA origami nanostructures. The well-defined, slower-migrating bands for the rod, rectangle, and icosahedron indicate successful folding of each structure as compared to the faster-migrating scaffold strand. The upward shift in mobility reflects increased molecular size and compactness due to base-pairing. The absence of lower-molecular-weight bands indicates effective removal of excess staple strands following PEG-based purification. C) AFM images highlighting the structural details of the various shapes.

## DNA Origami Diffusion Across Mucus: The Effect of Shape

To investigate the impact of the DNA origami shape on its diffusion across mucus, the three designs were evaluated in *ex vivo* porcine intestinal mucus using single-particle tracking (SPT) under a dSTORM microscope. SPT is a microscopy-based technique that enables a real-time observation of individual particle movements at a high spatial and temporal resolution, making it well-suited for studying diffusion behavior in complex biological environments.^27^ For visualization under dSTORM, DNA origami were labeled with the fluorophore ATTO 647. To follow their trajectories, we recorded videos over 10 s at 37 °C at 100 FPS (Figure 2A). Representative trajectories are depicted in Figure 2B, where we employed a color code to visually indicate the diffusion coefficient of each trajectory overtime. From these images, clear differences can already be observed among DNA origami shapes, where origami rods displayed trajectories with lower diffusion coefficient values, compared to rectangles and icosahedrons. This effect is more visible in Figure 2C, where we extracted and graphically represented different examples of trajectories from each shape, suggesting that the DNA origami nanorods remain largely confined within a specific region of the mucus, whereas both rectangles and icosahedra display higher area exploration. From the SPT of randomly selected particles (*N* = 50), we calculated their mean squared displacement (MSD) (Figure 2D) as previously reported.^41^ The MSD of particles serves as an indicator of their diffusive behavior. In this regard, the MSD exponent (α), obtained by fitting MSD as a function of time (⟨*r*2­(*t*)⟩ ∼ *t* ^α^), determines whether the particles exhibit normal diffusion (α ≈ 1), subdiffusion (α < 1), or superdiffusion/ballistic motion (α > 1).^42^ Interestingly, while both rectangles and icosahedrons displayed linear MSDs with an α value close to 1 (0.98 ± 0.02 and 1.037 ± 0.01, respectively), indicating Brownian diffusion, rods exhibited a slight subdiffusive behavior with an α value less than 1 (0.92 ± 0.02). This subdiffusion in rods suggests hindered translational motion, likely due to rotational constraints or interactions with the surrounding environment, which could be explained by their higher anisotropy compared to rectangles and icosahedrons. From the MSDs, we calculated the diffusion coefficients, where significant differences were observed (*p* < 0.05) among the three shapes, with values of 0.23 ± 0.01 μm^2^/s for rods, 0.44 ± 0.02 μm^2^/s for rectangles, and 0.75 ± 0.02 μm^2^/s for icosahedra (mean ± SEM) (Figure 2E). It is noteworthy that despite DNA nanorods and icosahedrons having a comparable theoretical volume (Supporting Table 1) their diffusion coefficient in mucus is significantly different. This can be explained by the observed differences in the hydrodynamic radius and diffusion behavior. This can be also observed for rectangles, whereas despite having a significant lower volume compared to icosahedrons, their diffusivity is not higher. We attribute this effect to the fact that both rectangles and rods have a higher anisotropy, resulting in higher hydrodynamic radius and constrained translational diffusivity. Overall, these results highlight the need to consider not only the theoretical particle sizes and volumes but also shape and anisotropy effects on the hydrodynamic size when studying nanoparticle diffusivity within mucus.

**Figure 2:**
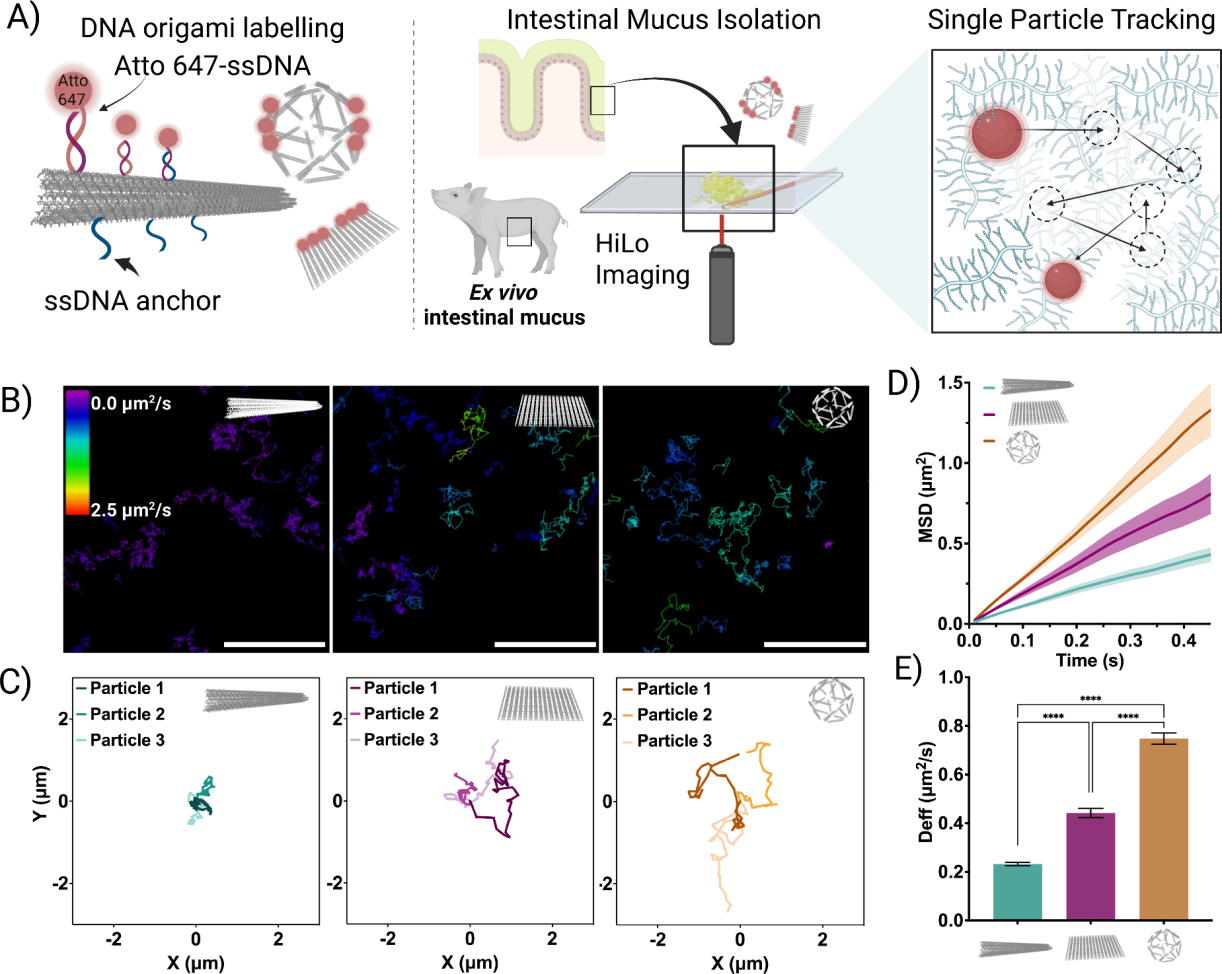
DNA origami tracking. A) Illustrative diagrams showing the tracking process of Atto 647-labeled DNA origami. Initially, *ex vivo* intestinal mucus is extracted from healthy piglets and incubated with DNA origami. The particles are then tracked by using HiLo microscopy. B) Top: Color-coded diffusion tracks for different shapes (scale bar 5 μm). C) Three representative tracks for each shape. D) Mean square displacement (MSD) analysis for different shapes in mucus. E) Diffusion coefficients extracted from the MSD analysis for the various shapes. Results are shown as the mean ± standard error of the mean (*N* = 50). Asterisks “****” denote statistically significant differences between groups, with *p* ≤ 0.0001.

Interestingly, our findings contrast with prior reports by Bao et al. and Yu et al.,^13,43^ which showed that rod-shaped nanoparticles diffuse faster than spherical ones in mucus. Yu et al. attributed this to shape-facilitated rotational dynamics under shear,^43^ while Bao et al. noted that flexible rods exhibited enhanced diffusion compared to both rigid rods and spheres.^13^ However, these studies involved smaller, synthetic, or polymer-based particles with surface chemistries designed to minimize mucoadhesion. In contrast, our DNA origami rods are larger, semiflexible, and highly negatively charged, which likely promotes electrostatic and steric interactions with the mucus mesh. These differences may account for the reduced diffusivity observed in our system and underscore the role of material properties in modulating shape-dependent transport.

## Diffusion Dynamics of Ligand-Functionalized DNA Origami

After characterizing the diffusion behavior of naked DNA origami, we aimed at investigating the effect of ligand functionalization. We selected the anti-EGFR antibody as a functional cargo due to its relevance in colorectal cancer therapeutics, where its delivery across mucus is highly desirable.^28^ To this end, anti-EGFR was conjugated to an ssDNA oligonucleotide using a click-chemistry approach. First, a dibenzocyclooctyne (DBCO) group was coupled to the antibody via an NHS-mediated coupling reaction, which was subsequently conjugated to a N_3_-modified ssDNA oligonucleotide. The resulting antibody–DNA conjugates were then incubated with the DNA origami nanostructures displaying complementary ssDNA sequences on their surfaces (Figure 3A). Gel electrophoresis and AFM analysis confirmed the successful conjugation of antibodies onto the DNA origami surface (Figures SI 2 and SI 5). Once assembled, DNA origami–antibody conjugates were tracked in mucus and analyzed, as previously described. Surprisingly, we observed that the rods conjugated with the antibody exhibited a significantly higher MSD slope (Figure 3B), compared to the unconjugated nanorods, indicating an enhanced diffusivity, which can be observed in Figure 3C, showing the diffusion coefficient values for the different samples. For DNA origami rods, the enhancement in diffusion was accompanied by a significant increase in the α value, suggesting a shift from subdiffusive behavior to Brownian diffusion. In contrast, for the rectangles and icosahedra, the opposite behavior was observed. These shapes displayed a decrease in their diffusivity, as indicated by a lower MSD and diffusion coefficient compared to the naked DNA origami structures, and the decrease in their α values suggested a shift from diffusive to subdiffusive behavior. This effect could be due to the geometric differences in the structures, leading to different degrees of anisotropy. The antibody used in our study is a positively charged protein, and previous research has shown that the asymmetric charge distribution and spatial configuration of charges on the surface of the carrier can significantly impact the rate of transport, enhancing their diffusivity in mucus.^44,45^ This suggests that both the antibody charge and its spatial arrangement on the DNA origami rods may play a crucial role in enhancing the diffusivity of the nanostructures, which is not observed for the other shapes. To confirm this hypothesis, we performed an additional experiment in which we substituted the antibodies with bovine serum albumin (negatively charged). Here, we observed that BSA conjugation also induced an increased diffusivity, though it was significantly less pronounced than with anti-EGFR. This suggests that enhanced diffusivity in mucus requires an asymmetric charge distribution (Figure SI 3). We conducted an additional control experiment, where we substituted mucus by glycerol. Interestingly, we observed that the diffusivity of antibody-conjugated nanorods was not significantly enhanced compared to naked nanorods (Figure SI 4B) However, this behavior differs in mucus, where factors such as shape and antibody distribution appear to play a more significant role in motility. This suggests that while size influences diffusion in simpler media the complexity of mucus requires consideration of additional parameters, such as surface properties and the spatial distribution of functional groups, to fully understand the particles’ movement. Therefore, we hypothesize that introducing asymmetric charge surfaces and carefully arranging these asymmetric charges can enhance the particle mobility in mucus. This is further confirmed by the notion that viruses, which present both positive and negative charges on their surfaces, may owe their rapid movement through mucus to the specific spatial arrangement of charges.^44,46^ To assess how antibody conjugation altered the surface charge properties of the DNA origami structures, we performed zeta potential measurements before and after functionalization (Figure SI 6), which revealed a clear reduction in surface charge for both the rod and icosahedral structures, consistent with successful functionalization and a shift toward a less negative surface. This aligns with the low isoelectric point of DNA and the higher isoelectric point of the anti-EGFR IgG1 antibody (7.5–9.0) under physiological conditions.^47,48^ Interestingly, no significant change was observed for the rectangle, likely due to its flat geometry and one-sided antibody placement, which may partially shield the surface charge during measurement.^49^ To better understand the shape-dependent changes in diffusion after antibody functionalization, we examined how the spatial arrangement of the ligands on each nanostructure influences interactions with mucus. Both the icosahedron and rectangle were functionalized asymmetrically on one side, but their distinct shapes likely modulate this effect. The icosahedron’s compact 3D geometry may promote local binding or rotational hindrance, while the rectangle’s flat surface may enhance steric interactions and contact with the mucus mesh, reducing mobility. In contrast, the rod’s elongated shape and more distributed curvature may enable streamlined diffusion and locally disrupt mucin entanglement without significantly increasing adhesion, contributing to the enhancement in mobility observed.

**Figure 3:**
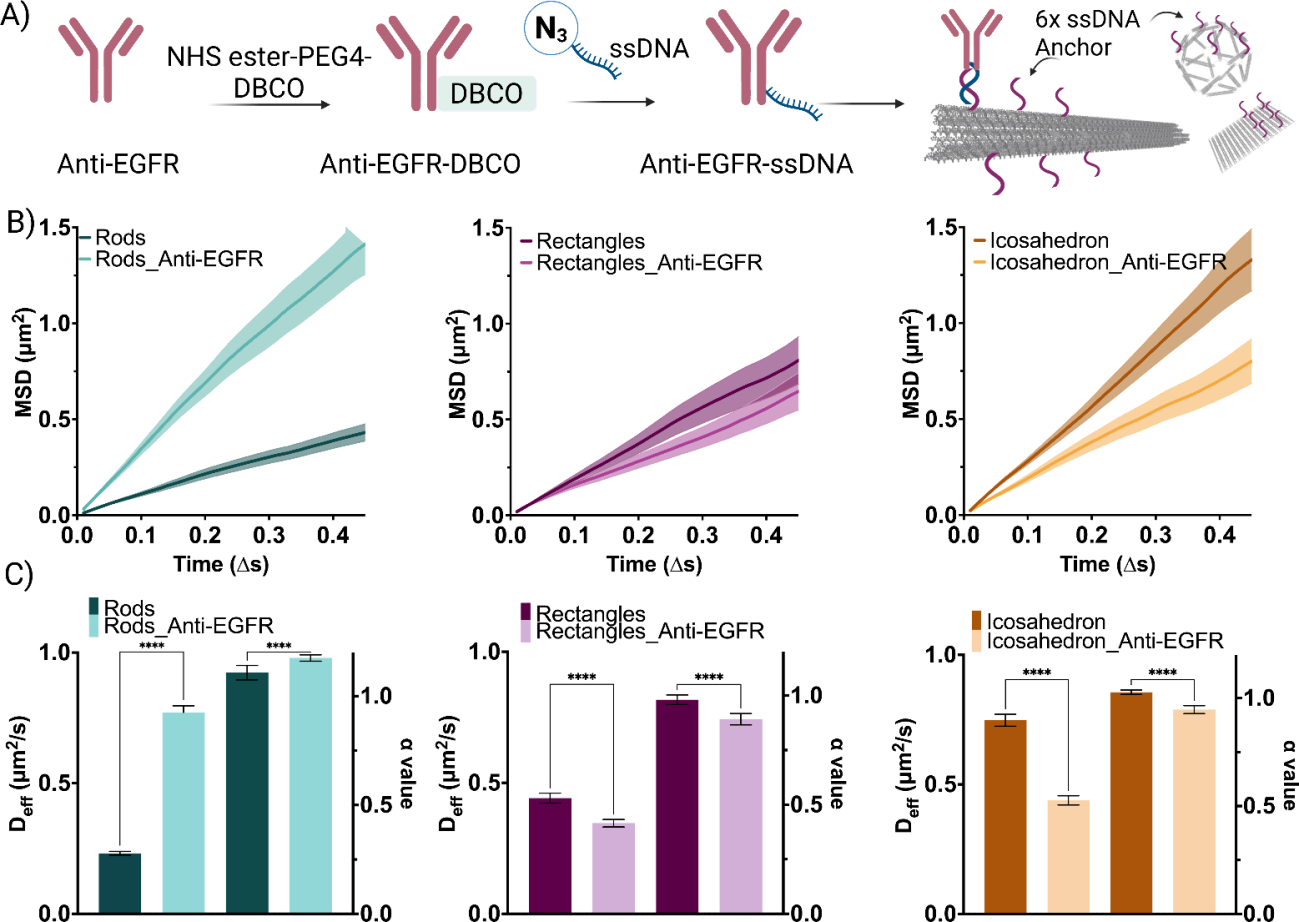
DNA origami conjugation. A) Illustrative diagrams depicting the functionalization of proteins via an NHS-mediated coupling reaction, followed by click chemistry with the azide-modified complementary handle on the DNA origami, and finally incubation with the DNA origami using a modified extended staple. B) Mean square displacement (MSD) analysis for different naked shapes and anti-EGFR conjugated DNA origami. C) Diffusion coefficients and alpha values extracted from the MSD analysis for the various shapes conjugated with the antibody. Results are shown as the mean ± standard error of the mean (*N* = 50). Asterisks “****” denote statistically significant differences between groups, with *p* ≤ 0.0001.

The differences in diffusion observed after antibody functionalization (particularly the contrasting behavior across different nanoparticle geometries) suggest that not only the presence of ligands but also their spatial distribution and density may significantly influence particle–mucin interactions. In particular, the Janus-like arrangement on 2D and 3D structures appears to introduce asymmetries that affect the mobility. These observations motivated a more targeted investigation into how varying ligand density and surface presentation modulate diffusion through mucus, which is addressed in the following section.

## Ligand Display Effects on DNA Origami Diffusion Across Mucus

To further investigate the effect of antibody functionalization on diffusion, we designed DNA origami shapes with 0, 3, 6, and 9 antibodies (Figure 4A) and monitored their diffusion across mucus and glycerol. AFM (Figure SI 5) and gel electrophoresis confirmed the successful conjugation of varying densities of anti-EGFR to the DNA origami (Figure 4B). In glycerol, as expected, we observed an inversely proportional effect, where the diffusion coefficient decreased at increasing antibody numbers functionalized on the DNA origami nanostructures. We attribute this to the increase in hydrodynamic size, which results in lower diffusivity (Figure SI 4C). By contrast, in mucus, we did not observe a consistent trend among shapes (Figure 4C). Our observations revealed that for modified rods the highest diffusion coefficient was achieved with six antibodies conjugated to the surface. These results support our hypothesis that not only the charge but also their distribution on the nanoparticle surface is a key determinant for their diffusivity across mucus and that this is dependent on the nanoparticle shape. In the case of 6× functionalized rods, we assume that this is the most anisotropic charge distribution, and therefore, the highest diffusivity is observed. By contrast, for rectangles, 3× antibody functionalization slightly increased their diffusivity, but at increasing antibody number, diffusivity decreased, reaching values even lower than the control, nonfunctionalized rectangles. In the case of icosahedrons, antibody functionalization not only improved diffusivity but also resulted in a significant reduction in all cases. In previous reports, transient, low-affinity interactions that allow particles to bind and release from mucin dynamically have previously been shown to increase the diffusivity of nanoparticles in mucus.^50,51^ Our results align with these observations, where functionalization with a low or medium density of positively charged antibodies may lead to dynamic, low affinity interactions with negatively charged mucin, whereas the functionalization with a higher antibody density results in strong electrostatic interactions with negatively charged mucin.

**Figure 4:**
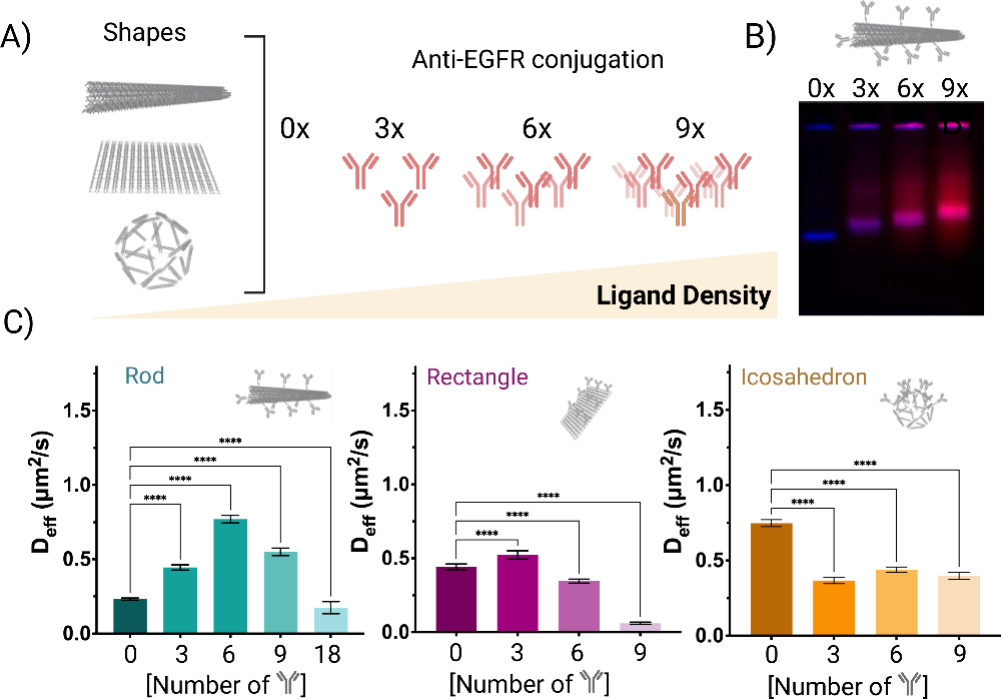
DNA origami density effect. A) Illustrative diagrams depicting the functionalization of different densities on the DNA origami. B) Gel electrophoresis results of I) DNA origami structure; II) DNA origami structure-3x aEGFR; III) DNA origami structure-6x aEGFR; and IV) DNA origami structure-9x aEGFR. C) Diffusion coefficients extracted from the MSD analysis for the different densities of antibodies conjugated to various shapes. Results are shown as the mean ± standard error of the mean (*N* = 50). Asterisks “****” denote statistically significant differences between groups, with *p* ≤ 0.0001.

Overall, our results indicate that there is a threshold where DNA origami functionalization with positively charged antibodies does not increase the diffusivity. These findings underscore the critical role of nanoparticle topological characteristics, including ligand charge, density, and particle shape, in governing mucus transport. A comprehensive understanding and precise control of these parameters will be essential for the rational design and optimization of nanoparticle-based drug delivery systems aimed at effectively traversing mucosal barriers effectively.

This work establishes DNA origami as a versatile and precisely tunable platform to decouple and systematically investigate the effects of nanoparticle topology (specifically shape, ligand functionalization, and ligand density) on mucus transport, while keeping material composition constant. Through high-resolution single-particle tracking, we captured the spatiotemporal dynamics of particle motion, yielding mechanistic insights into how anisotropy and surface presentation modulate the nanoparticle mobility through complex mucosal environments. Among the geometries studied, unmodified rod-shaped DNA origami exhibited the lowest diffusion coefficients; however, upon surface functionalization with a therapeutic antibody, their diffusivity increased markedly, surpassing that of both rectangle-shaped and icosahedral counterparts. This enhancement was maximized at an intermediate ligand density (six antibodies per particle), suggesting a geometry-specific optimum in the surface presentation. In contrast, rectangles and icosahedra showed only modest increases in diffusivity at low ligand densities (three antibodies per particle) with diminished transport at higher functionalization levels. These results highlight the nuanced, nonmonotonic relationship between ligand density and particle mobility and underscore the importance of shape-dependent optimization in the design of mucus-penetrating delivery systems. Collectively, our findings establish DNA origami as a powerful tool for elucidating transport mechanisms and guiding the rational design of next-generation nanocarriers for mucosal drug delivery.

## Supplementary Material



## Notes

Additional characterization of DNA origami nanostructures with DLS, AFM, gel electrophoresis, and detailed methodology (PDF)

## References

[1] Blanco E., Shen H., Ferrari M. (2015). Principles of Nanoparticle Design
for Overcoming Biological Barriers to Drug Delivery. Nat. Biotechnol..

[2] Mitchell M. J., Billingsley M. M., Haley R. M., Wechsler M. E., Peppas N. A., Langer R. (2021). Engineering
Precision Nanoparticles for Drug Delivery. Nat.
Rev. Drug Discov.

[3] Poon W., Kingston B. R., Ouyang B., Ngo W., Chan W. C. W. (2020). A Framework
for Designing Delivery Systems. Nat. Nanotechnol.

[4] Lai S. K., Wang Y.-Y., Wirtz D., Hanes J. (2009). Micro- and Macrorheology
of Mucus. Adv. Drug Deliv Rev..

[5] Murgia X., Loretz B., Hartwig O., Hittinger M., Lehr C.-M. (2018). The Role of Mucus on Drug Transport
and Its Potential
to Affect Therapeutic Outcomes. Adv. Drug Deliv
Rev..

[6] Ensign L. M., Cone R., Hanes J. (2014). Nanoparticle-Based Drug Delivery
to the Vagina: A Review. J. Controlled Release.

[7] Lai S. K., Wang Y.-Y., Hanes J. (2009). Mucus-Penetrating
Nanoparticles for
Drug and Gene Delivery to Mucosal Tissues. Adv.
Drug Deliv Rev..

[8] García-Díaz M., Birch D., Wan F., Nielsen H. M. (2018). The Role of Mucus
as an Invisible Cloak to Transepithelial Drug Delivery by Nanoparticles. Adv. Drug Deliv Rev..

[9] Mok Z. H. (2024). The Effect
of Particle Size on Drug Bioavailability in Various Parts of the Body. Pharmaceutical Science Advances.

[10] Guo M., Wei M., Li W., Guo M., Guo C., Ma M., Wang Y., Yang Z., Li M., Fu Q., Yang L., He Z. (2019). Impacts of Particle Shapes on the
Oral Delivery of Drug Nanocrystals: Mucus Permeation, Transepithelial
Transport and Bioavailability. J. Controlled
Release.

[11] Guo Y., Ma Y., Chen X., Li M., Ma X., Cheng G., Xue C., Zuo Y. Y., Sun B. (2023). Mucus Penetration of Surface-Engineered
Nanoparticles in Various PH Microenvironments. ACS Nano.

[12] Tollemeto M., Ursulska S., Welzen P. L. W., Thamdrup L. H. E., Malakpour-Permlid A., Li Y., Soufi G., Patiño Padial T., Christensen J. B., Hagner Nielsen L., van Hest J., Boisen A. (2024). Tailored Polymersomes
for Enhanced Oral Drug Delivery: PH-Sensitive Systems for Intestinal
Delivery of Immunosuppressants. Small.

[13] Bao C., Liu B., Li B., Chai J., Zhang L., Jiao L., Li D., Yu Z., Ren F., Shi X., Li Y. (2020). Enhanced Transport
of Shape and Rigidity-Tuned α-Lactalbumin Nanotubes across Intestinal
Mucus and Cellular Barriers. Nano Lett..

[14] Rothemund P. W. K. (2006). Folding
DNA to Create Nanoscale Shapes and Patterns. Nature.

[15] Douglas S. M., Dietz H., Liedl T., Högberg B., Graf F., Shih W. M. (2009). Self-Assembly of DNA into Nanoscale
Three-Dimensional Shapes. Nature.

[16] Dey S., Fan C., Gothelf K. V., Li J., Lin C., Liu L., Liu N., Nijenhuis M. A. D., Saccà B., Simmel F. C., Yan H., Zhan P. (2021). DNA Origami. Nature Reviews Methods Primers.

[17] Seeman N. C., Sleiman H. F. (2018). DNA Nanotechnology. Nat. Rev.
Mater..

[18] Wagenbauer K. F., Pham N., Gottschlich A., Kick B., Kozina V., Frank C., Trninic D., Stömmer P., Grünmeier R., Carlini E., Tsiverioti C. A., Kobold S., Funke J. J., Dietz H. (2023). Programmable Multispecific
DNA-Origami-Based T-Cell Engagers. Nat. Nanotechnol.

[19] Knappe G. A., Wamhoff E.-C., Bathe M. (2023). Functionalizing DNA Origami to Investigate
and Interact with Biological Systems. Nat. Rev.
Mater..

[20] Huang J., Jaekel A., van den Boom J., Podlesainski D., Elnaggar M., Heuer-Jungemann A., Kaiser M., Meyer H., Saccà B. (2024). A Modular
DNA Origami Nanocompartment for Engineering
a Cell-Free, Protein Unfolding and Degradation Pathway. Nat. Nanotechnol.

[21] Bila H., Paloja K., Caroprese V., Kononenko A., Bastings M. M. C. (2022). Multivalent Pattern Recognition through
Control of
Nano-Spacing in Low-Valency Super-Selective Materials. J. Am. Chem. Soc..

[22] Bastings M. M. C., Anastassacos F. M., Ponnuswamy N., Leifer F. G., Cuneo G., Lin C., Ingber D. E., Ryu J. H., Shih W. M. (2018). Modulation of the
Cellular Uptake
of DNA Origami through Control over Mass and Shape. Nano Lett..

[23] Mills A., Aissaoui N., Finkel J., Elezgaray J., Bellot G. (2023). Mechanical DNA Origami to Investigate
Biological Systems. Adv. Biol..

[24] Li S., Jiang Q., Liu S., Zhang Y., Tian Y., Song C., Wang J., Zou Y., Anderson G. J., Han J.-Y., Chang Y., Liu Y., Zhang C., Chen L., Zhou G., Nie G., Yan H., Ding B., Zhao Y. (2018). A DNA Nanorobot Functions as a Cancer
Therapeutic in Response to a Molecular Trigger in Vivo. Nat. Biotechnol..

[25] Tian T., Zhang T., Shi S., Gao Y., Cai X., Lin Y. (2023). A Dynamic DNA Tetrahedron Framework
for Active Targeting. Nat. Protoc.

[26] Zhang Q., Jiang Q., Li N., Dai L., Liu Q., Song L., Wang J., Li Y., Tian J., Ding B., Du Y. (2014). DNA Origami as an *In Vivo* Drug Delivery Vehicle for Cancer Therapy. ACS Nano.

[27] Wang W. X., Douglas T. R., Zhang H., Bhattacharya A., Rothenbroker M., Tang W., Sun Y., Jia Z., Muffat J., Li Y., Chou L. Y. T. (2024). Universal, Label-Free,
Single-Molecule Visualization of DNA Origami Nanodevices across Biological
Samples Using OrigamiFISH. Nat. Nanotechnol.

[28] Martinelli E., Ciardiello D., Martini G., Troiani T., Cardone C., Vitiello P. P., Normanno N., Rachiglio A. M., Maiello E., Latiano T., De Vita F., Ciardiello F. (2020). Implementing
Anti-Epidermal Growth Factor Receptor (EGFR) Therapy in Metastatic
Colorectal Cancer: Challenges and Future Perspectives. Annals of Oncology.

[29] Abramson A., Frederiksen M. R., Vegge A., Jensen B., Poulsen M., Mouridsen B., Jespersen M. O., Kirk R. K., Windum J., Hubálek F., Water J. J., Fels J., Gunnarsson S. B., Bohr A., Straarup E. M., Ley M. W. H., Lu X., Wainer J., Collins J., Tamang S., Ishida K., Hayward A., Herskind P., Buckley S. T., Roxhed N., Langer R., Rahbek U., Traverso G. (2022). Oral Delivery of Systemic
Monoclonal Antibodies, Peptides and Small Molecules Using Gastric
Auto-Injectors. Nat. Biotechnol..

[30] Angsantikul P., Peng K., Curreri A. M., Chua Y., Chen K. Z., Ehondor J., Mitragotri S. (2021). Ionic Liquids
and Deep Eutectic Solvents
for Enhanced Delivery of Antibodies in the Gastrointestinal Tract. Adv. Funct Mater..

[31] Van
Zundert I., Spezzani E., Brillas R. R., Paffen L., Yurchenko A., de Greef T. F. A., Albertazzi L., Bertucci A., Patino Padial T. (2025). Unveiling DNA Origami Interaction
Dynamics on Living Cell Surfaces by Single Particle Tracking. Small.

[32] Kempter S., Khmelinskaia A., Strauss M. T., Schwille P., Jungmann R., Liedl T., Bae W. (2019). Single Particle Tracking and Super-Resolution
Imaging of Membrane-Assisted Stop-and-Go Diffusion and Lattice Assembly
of DNA Origami. ACS Nano.

[33] Yildiz H. M., McKelvey C. A., Marsac P. J., Carrier R. L. (2015). Size Selectivity
of Intestinal Mucus to Diffusing Particulates Is Dependent on Surface
Chemistry and Exposure to Lipids. J. Drug Target.

[34] Boegh M., Nielsen H. M. (2015). Mucus as a Barrier to Drug Delivery
- Understanding
and Mimicking the Barrier Properties. Basic
Clin Pharmacol Toxicol.

[35] Stahl E., Martin T. G., Praetorius F., Dietz H. (2014). Facile and Scalable
Preparation of Pure and Dense DNA Origami Solutions. Angew. Chem., Int. Ed..

[36] Benson E., Mohammed A., Gardell J., Masich S., Czeizler E., Orponen P., Högberg B. (2015). DNA Rendering
of Polyhedral Meshes
at the Nanoscale. Nature 2015 523:7561.

[37] Spratt J., Dias J. M., Kolonelou C., Kiriako G., Engström E., Petrova E., Karampelias C., Cervenka I., Papanicolaou N., Lentini A., Reinius B., Andersson O., Ambrosetti E., Ruas J. L., Teixeira A. I. (2024). Multivalent Insulin
Receptor Activation Using Insulin-DNA Origami Nanostructures. Nat. Nanotechnol.

[38] Benson E., Mohammed A., Gardell J., Masich S., Czeizler E., Orponen P., Högberg B. (2015). DNA Rendering
of Polyhedral Meshes
at the Nanoscale. Nature.

[39] Hübner K., Raab M., Bohlen J., Bauer J., Tinnefeld P. (2022). Salt-Induced
Conformational Switching of a Flat Rectangular DNA Origami Structure. Nanoscale.

[40] Cremers G. A. O., Rosier B. J. H. M., Meijs A., Tito N. B., van Duijnhoven S. M. J., van Eenennaam H., Albertazzi L., de Greef T. F. A. (2021). Determinants of Ligand-Functionalized DNA Nanostructure-Cell
Interactions. J. Am. Chem. Soc..

[41] Patiño
Padial T., Del Grosso E., Gentile S., Baranda
Pellejero L., Mestre R., Paffen L. J. M. M., Sánchez S., Ricci F. (2024). Synthetic DNA-Based Swimmers Driven
by Enzyme Catalysis. J. Am. Chem. Soc..

[42] Ernst D., Köhler J., Weiss M. (2014). Probing the Type of Anomalous Diffusion
with Single-Particle Tracking. Phys. Chem. Chem.
Phys..

[43] Yu M., Wang J., Yang Y., Zhu C., Su Q., Guo S., Sun J., Gan Y., Shi X., Gao H. (2016). Rotation-Facilitated
Rapid Transport of Nanorods in Mucosal Tissues. Nano Lett..

[44] Li L. D., Crouzier T., Sarkar A., Dunphy L., Han J., Ribbeck K. (2013). Spatial Configuration and Composition of Charge Modulates
Transport into a Mucin Hydrogel Barrier. Biophys.
J..

[45] Leal J., Dong T., Taylor A., Siegrist E., Gao F., Smyth H. D. C., Ghosh D. (2018). Mucus-Penetrating Phage-Displayed
Peptides for Improved Transport across a Mucus-like Model. Int. J. Pharm..

[46] Cone R. A. (2009). Barrier
Properties of Mucus. Adv. Drug Deliv Rev..

[47] Bumbaca D., Boswell C. A., Fielder P. J., Khawli L. A. (2012). Physiochemical and
Biochemical Factors Influencing the Pharmacokinetics of Antibody Therapeutics. AAPS Journal.

[48] Jain T., Sun T., Durand S., Hall A., Houston N. R., Nett J. H., Sharkey B., Bobrowicz B., Caffry I., Yu Y., Cao Y., Lynaugh H., Brown M., Baruah H., Gray L. T., Krauland E. M., Xu Y., Vásquez M., Wittrup K. D. (2017). Biophysical Properties
of the Clinical-Stage Antibody
Landscape. Proc. Natl. Acad. Sci. U. S. A..

[49] Öztürk K., Kaplan M., Çalıs S. (2024). Effects of Nanoparticle Size, Shape,
and Zeta Potential on Drug Delivery. Int. J.
Pharm..

[50] Sun X., Abioye R. O., Okagu O. D., Udenigwe C. C. (2022). Peptide-Mucin Binding
and Biosimilar Mucus-Permeating Properties. Gels.

[51] Schaefer A., Lai S. K. (2022). The Biophysical
Principles Underpinning Muco-Trapping
Functions of Antibodies. Hum Vaccin Immunother.

